# Clinical, Radiological, and Surgical Risk Factors for Endoscopic Anastomotic Recurrence Following Surgery in Crohn’s Disease

**DOI:** 10.3390/jcm13226669

**Published:** 2024-11-06

**Authors:** Laura Maria Minordi, Franco Sacchetti, Domenico Balzano, Rossella Maresca, Francesca Bice D’Angelo, Luigi Larosa, Davide Carano, Lucrezia Laterza, Daniela Pugliese, Paola Caprino, Angelo Eugenio Potenza, Franco Scaldaferri, Luigi Sofo, Evis Sala

**Affiliations:** 1 UOC di Radioterapia Oncologica ed Ematologia Dipartimento di Diagnostica per Immagini, Fondazione Policlinico Universitario “A. Gemelli” IRCCS, 00168 Rome, Italy; lauramaria.minordi@policlinicogemelli.it (L.M.M.); francescabice.dangelo01@icatt.it (F.B.D.); luigi.larosa@policlinicogemelli.it (L.L.); davide.carano@guest.policlinicogemelli.it (D.C.); evis.sala@policlinicogemelli.it (E.S.); 2 UOC di Chirurgia Addominale, Dipartimento di Scienze Mediche e Chirurgiche, Fondazione Policlinico Universitario “A. Gemelli” IRCCS, 00168 Rome, Italy; domenico.balzano01@icatt.it (D.B.); paola.caprino@policlinicogemelli.it (P.C.); angeloeugenio.potenza@policlinicogemelli.it (A.E.P.); luigi.sofo@policlinicogemelli.it (L.S.); 3 UOS Malattie Infiammatorie Croniche Intestinali, Centro Malattie Apparato Digerente (CeMAD), Dipartimento di Scienze Mediche e Chirurgiche, Fondazione Policlinico Universitario “A. Gemelli” IRCCS, 00168 Rome, Italy; rossella.maresca12@gmail.com (R.M.); lucrezia.laterza@policlinicogemelli.it (L.L.); daniela.pugliese@policlinicogemelli.it (D.P.); franco.scaldaferri@policlinicogemelli.it (F.S.); 4 Dipartimento di Medicina e Chirurgia Traslazionale, Università Cattolica del Sacro Cuore, 00168 Rome, Italy

**Keywords:** Crohn’s disease, postoperative recurrence, endoscopic recurrence, radiological recurrence

## Abstract

**Objective:** This study investigated the radiological, clinical, and surgical factors linked to the risk of endoscopic recurrence following ileocolic resection for Crohn’s disease. **Materials and Methods:** We conducted a retrospective analysis of data from all patients who underwent primary ileocecal resection for Crohn’s disease in a single colorectal unit between 2004 and 2020. We analyzed the potential risk factors subdivided by the clinical, radiological, and surgical factors associated with morphological recurrence, as detected by endoscopy within 2 years after surgery. Cox regression was employed to ascertain the risk factors associated with such recurrence. **Results:** In total, 63 patients were included, and 24 (38%) had endoscopic recurrence. The age of the patient at the time of surgery was identified as a significant clinical factor associated with the risk of recurrence (HR: 1.04; *p* = 0.003), indicating that the probability of recurrence increases by 1% as the surgical age increases each year. The radiological factors associated with an increased risk of recurrence included localization in the distal ileum (HR: 3.526; *p* = 0.015), the number of pathological small-bowel segments affected by the disease (HR: 1.15; *p* = 0.004), and the total length of the pathological intestinal segment (HR: 1.002; *p* = 0.014). The presence of granulomas (HR: 6.003; *p* = 0.004) and the length of the resected bowel (HR: 1.01; *p* = 0.003) were surgical factors associated with a higher risk of recurrence. **Conclusions:** This study delineated several clinical, radiological, and surgical factors that serve as predictors for the endoscopic recurrence of Crohn’s disease after surgery.

## 1. Introduction

Up to 80% of patients with Crohn’s disease (CD) undergo surgery during their lifetime, despite improvements in medical therapy with the introduction of new drugs [1]. Moreover, there has been important progress in surgery, with a tendency to carry out more limited surgical demolitions and/or stricture-plastic [2].

The pre-surgical evaluation of patients with CD is complex and requires a multidisciplinary approach with gastroenterologists, surgeons, and radiologists. Moreover, once the surgery has been performed, the clinical course of the patient is variable, with frequent postoperative relapses or recurrence, which may require a new surgery [3]. According to ECCO 2016 [4], the term “relapse” refers to the appearance of symptoms in a patient in clinical remission, either spontaneously or after medical therapy, and this must be confirmed by laboratory tests, radiological tests, or endoscopy. The term “recurrence” must be used to indicate the appearance of lesions after surgical therapy, and it is divided into morphological and clinical recurrence. Clinical recurrence consists of the appearance of symptoms in a patient undergoing surgery with complete resection of macroscopic disease. Morphological recurrence consists of the appearance of new lesions after complete resection of macroscopic disease and usually affects the terminal ileum before the anastomosis. Morphological recurrence is identified via endoscopy, radiology, or a new surgery.

In the literature, there are some studies that have evaluated the predictive risk factors of recurrence on the basis of clinical and laboratory parameters [5], while the role of imaging has not been thoroughly investigated [6], or it has been evaluated only in clinical studies [7]. The identification of patients with a high risk of recurrence is important in order to plan the correct therapeutic strategy after surgery.

The aim of this study was to evaluate the radiological factors that can predict the onset of endoscopic recurrence after surgery in association with clinical and surgical factors. The secondary objective was the evaluation of the survival time to the onset of recurrence after surgery.

## 2. Materials and Methods

### 2.1. Study Population and Data Source

We performed a monocentric retrospective study of a cohort of patients with known Crohn’s disease, which was submitted and approved by our Ethics Committee.

In the surgery department, electronic patient records were interrogated to identify patients who had undergone ileocolic resection for the first time between January 2014 and December 2020. Patients with a postoperative diagnosis of ulcerative colitis or unclassified inflammatory disease, previous surgical intestinal resection, postoperative diagnosis of tuberculosis, and histological evidence of invasive malignancy were excluded from this study. Patients of <18 years were also excluded. The diagnosis of Crohn’s disease was confirmed upon review of the medical records based on standard clinical, radiological, endoscopic, and histological reports.

For each patient, data were extracted from their medical records in the gastroenterology department to include demographic information, the Crohn’s disease clinical setting, operative and histological data, and medication history, while radiological exams (CT enterography, abdomen CT, and MR enterography) were evaluated in the radiological department.

All patients had either an abdomen CT or MR enterography performed within 6 months before surgery and underwent endoscopy within 2 years after the surgery.

The CT and MR enterography were independently evaluated by 3 radiologists with at least 10 years of abdominal radiology experience.

According to ECCO 2016 [4], we stated morphological recurrence in case of new CD lesions after complete resection of macroscopic disease, detected by endoscopy within 2 years.

In this study, the risk factors were divided into three groups: clinical, radiological, and surgical factors.

### 2.2. Clinical Evaluation

The clinical potential risk factors for the development of postoperative recurrence evaluated in this study were (1) age at the moment of diagnosis; (2) age at the moment of surgery; (3) gender; (4) the duration of the disease; (5) perianal disease; (6) a habit of smoking; (7) the presence and type of extra-intestinal manifestations; and (8) the type of medical therapy performed before surgical resection.

### 2.3. Technique and Radiological Evaluation

The radiological examinations performed on the patients within 6 months before surgery were evaluated. In the presence of multiple studies, the examination closest to the surgery was considered.

Both the CT and MR-E exams were performed according to standardized protocols used in our department [8,9].

In particular, the CT enterography (CT-E) exams were performed after injection of iodinated contrast medium and acquired 75 s after intravenous injection of 100–130 mL of iodinated contrast agent, administered in two consecutive boluses, the first bolus at a rate of 1.5 mL/sec (comprising 1/3 of the total amount of iodinated contrast agent given to the patient), and the second bolus at a rate of 3 mL/second (covering the remaining 2/3 of the total amount of iodinated contrast agent given to the patient).

The MR enterography (MR-E) exams were performed before and after intravenous administration of paramagnetic contrast medium using the following sequences: single-shot T2-weighted and balanced steady-state free precession (bSSFP), and T2-weighted fat-suppressed, multiphase 3D T1-weighted fat-suppressed post-contrast images. Diffusion-weighted imaging (with values of 0–800 s/mm^2^) sequences were performed but not evaluated in this study. Enhanced sequences were performed in the arterial, venous, and tardive phases.

In both the CT-E and MR-E, all patients had drunk a polyethylene glycol solution (1.5–2 L) in the 30 min preceding the examination. An anticholinergic was always administered in the MR-E exams, while it was optional in the CT-E exams, so it was not infused in all patients.

Abdomen CT was performed before and after injection of iodinated contrast medium and acquired 75 s after intravenous injection of 100–130 mL of iodinated contrast agent.

The following data were reported in an Excel sheet (Table 1):

1. Type of radiological examination (CTE; MR-E, abdomen CT);
2. Site of pathology;
3. Number of small-bowel pathological loops;
4. Characteristics of small-bowel pathological loops;

Length of each small-bowel pathological loop.

**Figure 1:**
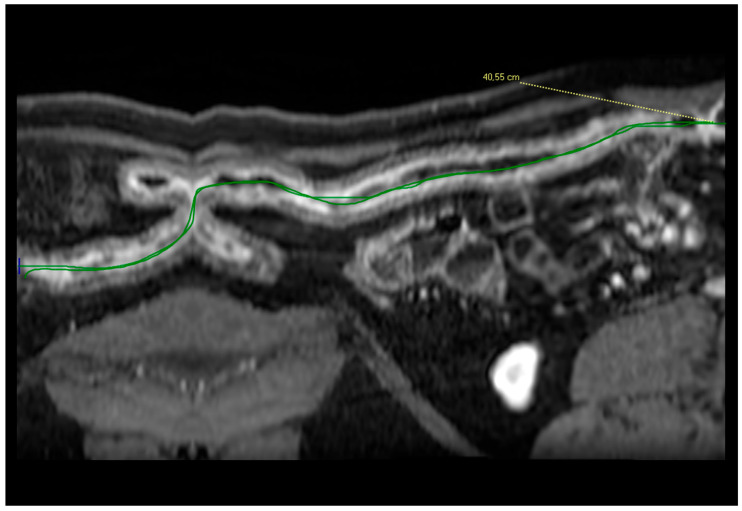
Post-processing reconstruction of the images enabled us to generate a linear view of the small bowel, allowing for the measurement of its length.

The measurement of the extent was performed in both CT and MRI, using vessel analysis software. The CT or MR images were reconstructed with post-processing “multiplanar reformatting” (MPR), and a specific software (Vue PACS Carestream V12) was applied to create a virtual image that permitted us to have a tubular vision of the small-bowel loops (Figure 1) [10]. This applied to the sum of inflammation in the discontinuous loops. The length of each pathological loop and the sum of the lengths of the pathological segments were calculated in patients with more than one small-bowel pathological loop.

Based on the signs described above, at the end of the radiological evaluation, a radiological judgment was expressed on the type of disease: active inflammatory, fistulizing, or fibrostenotic. In particular, the fistulizing subtype was identified in the presence of fistulas, sinus tracts, or abscesses.

**Table 1:** Radiological findings evaluated in CT/MR-E.

Type of Radiological Examination	MR Enterography or Abdomen CT or CT Enterography
Site of pathological loops	Proximal jejunum, distal jejunum, proximal ileum, distal ileum, last ileal loop, appendix, ascending colon, transverse colon, descending colon, sigmoid colon, and rectum
Number of small-bowel pathological loops	Counting as pathological any segment with radiological signs of CD separated from another lesion by a normal intestinal loop
Characteristics of small-bowel pathological loops	Mural thickening: a wall thickness of more than 3 mm; Mucosal ulcers: deep depressions in the mucosal surface; Bowel wall enhancement: stratified in the active inflammatory subtype (intense enhancement of the mucosa and serosa and low signal intensity in the submucosa) or homogeneous in the fibrostenotic subtype; Halo fatty sign: the presence of fatty signals in the submucosa; Engorgement of the vasa recta: hyperemia of the near mesentery; Stenosis: upstream dilatation with a loop caliber greater than 2 cm; Fibrofatty proliferation: excess of mesenteric fat; Sinus tracts: wall defects that extend outside the intestinal wall but have no connection to an epithelialized structure, such as adjacent organs or skin; Fistulas: communication with a near structure, e.g., entero-enteric, entero-colic, entero-cutaneous, and entero-vesicular fistulas; Abscesses: capsulated fluid collection near pathological loop, which could contain air; Lymph node enlargement: a short diameter of greater than 1 cm; Others: involvement of other structures, such as the colon, appendix, genital organs, etc.; In patients studied by MRI: bowel wall edema (a hyperintense signal in the wall compared with the skeletal muscle in T2-weighted sequences) and diffusion restriction in DWI (diffusion weighted imaging) sequences.
Length of each small-bowel pathological loop	In the presence of more than one pathological loop: the loop with greater extension; In the presence of more than one loop affected by pathology: the sum of the extension of the intestinal loops in which these segments are evident.
Type of disease	Active inflammatory; Fistulizing; Fibrostenotic.

### 2.4. Surgical and Postoperative Evaluation

In an Excel sheet, we reported the type of surgery (ileocecal resection, ileocolic resection, or small-bowel resection); traditional (LPT) or laparoscopic surgery; emergency or elective surgery; the length of the intestinal segment removed (cm); the presence of granulomas in the specimen; histological signs of Crohn’s disease in the resection margins; the type of anastomosis (stapled anastomosis or hand-sewn anastomosis); and ileostomy/colostomy.

In an Excel sheet, we reported postoperative complications. The Clavien–Dindo system was used to evaluate the grade of postsurgical complications [11].

### 2.5. Statistical Analysis

The clinical and demographic characteristics of the enrolled sample are shown with the descriptive statistics. Particularly, the quantitative variables are represented as means ± standard deviation (SD) or medians (IQR), as appropriate, according to the results of the Shapiro–Wilk test.

The qualitative variables are described in terms of absolute and percentage frequencies. The chi-squared and/or Fisher’s exact test were used to compare the categorical variables. Student’s *t*-tests and the Mann–Whitney test were used to compare the quantitative unpaired data, as appropriate.

Kaplan–Meier plots and log-rank tests were used to estimate and compare two or more survival curves. Cox proportional hazard regression models were applied to the estimated hazard ratios (HRs) and the effects of the predictor factors upon survival for their prognostic relevance. A *p*-value of <0.05 was considered statistically significant. The statistical analysis was assessed using the R software, version 4.4.0 (R Core Team (2024)).

## 3. Results

### 3.1. Baseline Characteristics

Two hundred and twenty-nine patients were identified for this study. Of these, 109 were excluded because they did not have radiological imaging within 6 months before surgery. Of the remaining 120, 57 patients were excluded because they had not had endoscopic exams in the two years following surgery or were lost to follow-up.

Of the remaining 63 patients who met all the criteria, 35 (55%) patients were evaluated by MR-E, 13 (21%) by CT-E, and 15 (24%) by abdomen CT.

All patients underwent endoscopy in the two years following surgery. Endoscopic recurrence of the disease was found in 24 patients (38%).

### 3.2. Clinical Evaluation

The clinical factors are described in Table 2 and Table 3, particularly the following:

(a) Age at diagnosis and at the time of surgery: the mean age was 32 years at the time of diagnosis (range: 10–69 years ±16.1) and 40 years at the time of surgery (range: 18–70 ±15.4 years).
(b) Gender: 35 males (56%) and 28 females (44%).
(c) Duration: the duration of the disease ranged from 0.5 to 46 years, with a mean of 8.4 years (±9.2 years).
(d) Perianal disease: perianal disease was present in 4 (6%) patients.
(e) A habit of smoking was present in 11 (17%) patients.

Extra-intestinal disease was present in 12 (9%) patients.

Age at the time of surgery was the only statistically significant factor (HR: 1.04; CI 1.01–1.06; *p* = 0.003) (Table 4).

In other words, the probability of having a recurrence increased by 1% for every year of increase in age. The other clinical factors were not statistically significant factors. In particular, age at diagnosis and the type of medical therapy were not statistically significant factors (Table 4).

**Table 2:** Demographic characteristics of patients.

	Minimum (Years)	Maximum (Years)	Mean (Years)	Standard Deviation
Age at diagnosis	10.7	69	31.9	16.1
Age at the moment of surgery	18	70	39.9	15.4
Duration of the disease	0.5	46	8.4	9.2

**Table 3:** Clinical factors and medical treatment of patients.

	Number of Patients	Percentage (%)
Male	35	55.6
Female	28	44.4
Smoking habit	11	17.5
Extra-intestinal manifestations:	12	9
Dermatological	2	3.2
Rheumatological	10	15.9
Perianal disease	4	6.3
Medical therapy:		
- Anti-TNF ^a^	11	17.5
- Anti-IL23 ^b^	2	3.2
- Anti-integrin	5	7.9
- Immunosuppressive drugs	5	7.9
- Budesonide	12	19.7
- Systemic corticosteroids	2	3.2
- 5-ASA ^c^	14	23

^a^ Anti-tumor necrosis factor; ^b^ anti-interleukin 23; ^c^ 5-acetylsalicylic acid.

**Table 4:** Statistical descriptive results of enrolled sample.

	HR (95% IC, *p*-Value)
Clinical factors:	
- Age at surgery (for each increment of one year)	1.04 (1.01–1.06, *p* = 0.003)
- Age at diagnosis	1.02 (0.99–1.04, *p* = 0.135)
- Gender	0.80 (1.24–0.34, *p* = 0.601)
- Duration of disease	1.07 (1.02–1.12, *p* = 0.051)
- Perianal disease	NA
- Habit of smoking:	
No	1.67 (0.63–4.36, *p* = 0.295)
Yes	3.08 (0.86–11.04, *p* = 0.083)
- Extra-intestinal manifestations	1.05 (0.41–2.67, *p* = 0.905)
- Anti-TNF ^a^	0.91(0.31–2.63, *p* = 0.860)
- Anti-IL22 ^b^	1.51 (0.19–11.35, *p* = 0.691)
- Anti-integrin	0.96 (0.22–4.21, *p* = 0.962)
- Immunosuppressive drugs	1.14 (0.25–5.02, *p* = 0.866)
- Budesonide	1.08 (0.27–4.27, *p* = 0.905)
- Systemic corticosteroids	0.76 (0.09–5.92, *p* = 0.796)
- 5-ASA ^c^	1.46 (0.48–4.38, *p* = 0.499)
Radiological factors:	
- Small-bowel thickening	0.96 (0.85–1.09, *p* = 0.525)
- Mucosal ulcers	1.12 (0.50–2.52, *p* = 0.771)
- Stratified CE	NA
- Halo fatty sign	NA
- Engorgement of vasa recta	0.44 (0.05–3.29, *p* = 0.425)
- Stenosis (lumen diameter)	0.78 (0.56–1.08, *p* = 0.137)
- Caliber of upstream loop	1.02 (1.00–1.04, *p* = 0.088)
- Sinus tracts	0.701 (0.31–1.63, *p* = 0.418)
- Fistulas	0.807 (0.35–1.82, *p* = 0.609)
- Abscesses	0.71 (0.24–2.08, *p* = 0.529)
- Lymph nodes	2.34 (0.54–9.98, *p* = 0.251)
- Localization in distal ileum	3.52 (1.19–9.03, *p* = 0.015)
- Number of small-bowel pathological loops	1.15 (1.05–1.27, *p* = 0.004)
- Length of small-bowel pathological loops	1.001(0.99–1.004, *p* = 0.452)
- Sum of small-bowel pathological loops	1.002 (1.00–1.54, *p* = 0.014)
- MRI hyperintensity	NA
- MRI restriction	NA
Surgical factors:	
- Laparotomic surgery	2.17 (0.96–4.86, *p* = 0.061)
- Length of resected intestine	1.01 (1.00–1.02, *p* = 0.003)
- Presence of granulomas	6.00 (1.05–1.27, *p* = 0.004)
- Histological involvement of resection margins	0.77 (0.32–1.78, *p* = 0.536)
- Hand-sewn anastomosis	6.67 (0.89–5.02, *p* = 0.033)
Postoperative evaluation:	
- Complications	0.54 (0.21–1.38, *p* = 0.204)

^a^ Anti-tumor necrosis factor; ^b^ anti-interleukin 23; ^c^ 5-acetylsalicylic acid.

### 3.3. Radiological Evaluation

The radiological exams identified 115 small-bowel loops affected by pathology. For each patient, the number of pathological small loops varied from 1 to 20 (medium: 3).

Table 5 shows the frequency distribution of the number of segments in our study.

The most frequent localizations were the last ileal loop (62 segments (98%)) and the distal ileum (41 segments (65%)). Even if the radiological evaluation was aimed at studying only the small intestine, we reported each colonic localization appreciable with the method, identifying 41 colonic segments affected by pathology. For each patient, the number of colonic localizations varied from zero to four (medium: one).

Concerning the radiological findings, small-bowel wall thickening was present in all patients, stenosis in 60 (95%), and intestinal dilatation in 32 (51%).

In Table 6, the values of the wall thickening, lumen diameter, and pre-stenotic loop caliber in all patients are reported.

In Table 7, the frequency values of the radiological signs found in this study are reported. In particular, fistulas were found in 27 patients (48%) and sinus tracts in 23 (36%). In six patients, sinus tracts were present without associated fistulas.

In patients who underwent MR-E (35 patients), an increased intramural T2 signal was found in all patients (100%) and restricted diffusion in DWI in 30 (86%) (Table 6).

Table 8 reports the length of each small-bowel pathological loop. The sum of the pathological segments is reported for patients with more than one small-bowel pathological loop.

On the basis of the signs described above, active inflammatory disease was found in 26 (41%) patients, fistulizing in 33 (52%) patients, and fibrostenotic in 4 (6%) patients.

As complications, occlusion was present in 8 (13%) patients. None of the patients reported perforation or bleeding.

**Figure 2:**
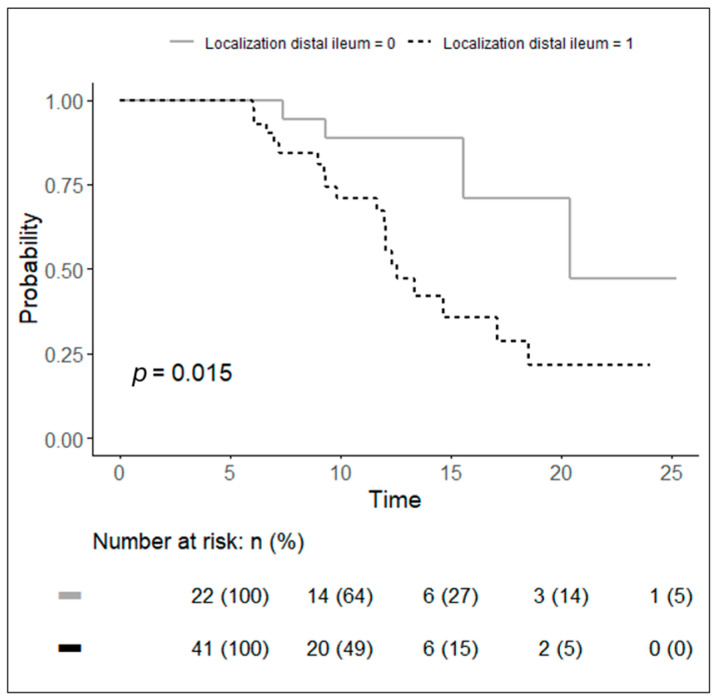
Kaplan–Meier curves of probability to have a recurrence in patients with radiological distal ileum localization (dotted black line) and patients who did not have radiological localization in the distal ileum (continuous grey line).

Localization in the distal ileum (HR: 3.526; *p* = 0.015; 1.19–9.03) and the number of pathological small-bowel loops (HR: 1.15; 1.05–1.27; *p* = 0.004) were statistically significant radiological factors (Table 3). In other words, patients who had localization in the distal ileum were 3.5 times more likely to develop recurrence than patients who did not have localization in the distal ileum (Figure 2), while the probability of recurrence increased by 1% for each increase in the number of small-bowel loops.

In patients with localization in the distal ileum, the median time from ileocolic resection to clinical postoperative recurrence was 12.5 months.

The sum of small-bowel pathological loops was also a statistically significant factor (HR. 1.002; IC: 1.00–1.54; *p* = 0.014). In other words, the probability of having a recurrence increased by 1% for every increase in cm. The other radiological factors were not statistically significant factors (Table 3).

### 3.4. Surgical Evaluation

Ileocecal resection was performed in 48 (76%) of patients, and the laparoscopic technique was used for 46 patients (73%). The other surgical factors are described in Table 9. The length of the resected small bowel had a minimum of 3 cm, a maximum of 175 cm, and a mean of 40.7 cm.

**Figure 3:**
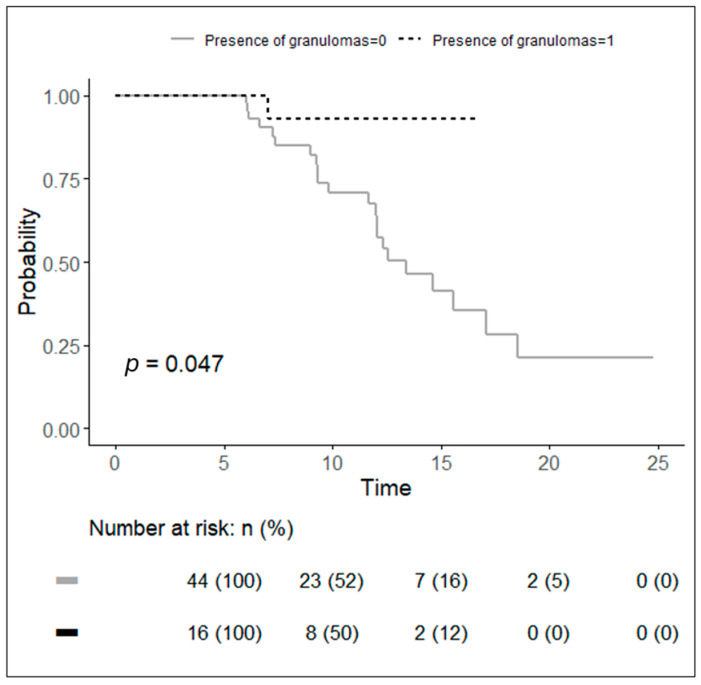
Kaplan–Meier curves of probability to have a recurrence in patients with the presence of granulomas in the specimens (dotted black line) and patients without the presence of granulomas (continuous grey line).

The length of the resected intestine (HR: 1.01; 1.00–1.02; *p* = 0.003) was a statistically significant factor. In other words, the probability of recurrence increased by 1% for each cm of increase in the length (Table 4). The presence of granulomas (HR: 6.003; IC: 1.05–1.27; *p* = 0.004 (Figure 3)) and hand-sewn anastomosis (HR: 6.67; 0.89–5.02; *p* = 0.033) were also statistically significant factors (Table 4). This means that the probability of recurrence was six times more likely to be in the presence of granulomas and almost seven times more likely to be in the case of hand-sewn anastomosis.

In patients with hand-sewn anastomosis, the median time from ileocolic resection to clinical postoperative recurrence was 14.6 months, while it was 13.4 months in patients with granulomas.

The other surgical factors were not statistically significant factors (Table 4).

### 3.5. Postsurgical Evaluation

After surgery, 22 (35%) patients had complications (Table 10). The most frequent complication was melena/rectorrhagia in 9 (14%) patients.

The presence of complications was not a statistically significant factor for recurrence (Table 4).

## 4. Discussion

### 4.1. Definition of Recurrence

In the literature, scientific papers are not homogeneous regarding the method of evaluation and identification of postoperative recurrence. In 2014, Li et al. [6] evaluated patients with endoscopic recurrence. In 2015, De Cruz, P. et al. [12] evaluated patients with clinical relapse, defined as the recurrence of Crohn’s disease symptoms leading to hospitalization or therapeutic modifications. In 2021, Navaratne et al. [7] distinguished between clinical relapse and symptomatic relapse: clinical relapse was defined as the recurrence of Crohn’s disease symptoms leading to hospitalization or therapeutic modifications, according to De Cruz [12], while postoperative relapse was defined as symptomatic when clinical recurrence was confirmed by the presence of recurrence in endoscopic and/or radiological examinations. In 2021, in a paper by Otzgur et al. [13], recurrence was evaluated based on the Crohn’s Disease Activity Index or endoscopic findings. In 2016, the ECCO guidelines [4] standardized the terminology to be used in the definition of postoperative recurrence. According to the ECCO guidelines, we chose to evaluate patients with morphological recurrence identified by endoscopy within 2 years before surgery, with the aim of verifying the roles of clinical, surgical, and radiological factors in postoperative recurrence.

### 4.2. Clinical Factor

In 2015, Fornaro et al. [14] published a clinical narrative review and analyzed the roles of different factors in the occurrence of postoperative recurrence in patients surgically treated for CD. The paper aimed to determine which of these factors had been proven in the literature to have a predictive role. The authors underlined the discordant results reported in the literature for most of the clinical factors, even if some of them seemed to be correlated with a greater risk of recurrence. In particular, smoking was reported to be the most statistically significant among the clinical factors. On the contrary, in our study, smoking was not a statistically significant factor.

In 2021, in a study by Navaratne et al. [7], a univariate analysis of the Montreal classification A1 indicated that a Crohn’s disease diagnosis age of <17 years appeared to be associated with an increased risk of symptomatic anastomotic postoperative recurrence. In our study, age at diagnosis was not a statistically significant factor. On the contrary, we found age at the time of surgery (HR: 1.04; CI: 1.01–1.06; *p* = 0.003) to be the only statistically significant clinical factor. To the best of our knowledge, this factor has not been evaluated in previous studies.

The other clinical factors were not statistically significant.

### 4.3. Radiological Factors

In our study, localization in the distal ileum was a statistically significant radiological factor (*p* = 0.015; HR: 3.526; 1.19–9.03). This means that patients who have localization in the distal ileum in radiological exams are more likely to develop recurrence than patients who do not have localization in the distal ileum. In patients with localization in the distal ileum, the median time from ileocolic resection to clinical postoperative recurrence was 12.5 months. Colic localization was not a statistically significant factor. However, in the literature, not all authors agree with the importance of intestinal localization. In fact, for some authors, colic localization seems to be associated with a lower recurrence rate than jejunal and ileal disease [15], while for others [16], localization of the disease in the colon is a predictive factor for recurrence.

In the literature, mesenteric hypertrophy is described in some studies as an indicator of a complicated course of Crohn’s disease [6,17]. Li et al. [6] used computed tomography to measure the subcutaneous fat area and visceral fat area (VFA) and defined the mesenteric fat index (MFI) as the ratio of the VFA to the subcutaneous fat area. The authors found that a high VFA value is predictive of postoperative recurrence of Crohn’s disease, so it plays a clinical role in optimizing prophylaxis for each patient. Navaratne et al. [7] evaluated the mesenteric fat index and the presence of fibrofatty proliferation in CT/MRI examinations carried out before surgery, and no association was found between the visceral fat area (VFA) or mesenteric fat index (MFI) and postoperative recurrence. In our study, we evaluated the presence of fibrofatty proliferation, and we found it not to be a statistically significant factor in predicting recurrence.

Finally, a significant radiological factor was the number of pathological small-bowel loops affected by pathology (HR: 1.15; 1.05–1.27; *p* =0.004). To the best of our knowledge, this factor has not been evaluated in previous studies.

### 4.4. Surgical Evaluation

In “The Second European Evidence-Based Consensus on the Diagnosis and the Management of Crohn’s Disease: Special Situations” [16], a penetrating behavior of the disease, extensive small-bowel resection, and prior intestinal surgery were also predictive factors for postoperative recurrence. In our study, we evaluated patients who underwent surgery for the first time; therefore, we were not able to make any judgments regarding this factor, and the presence of penetrating disease was not a statistically significant factor.

Concerning disease extension, Navaratne et al. [7] evaluated the extension of disease based on the extent of macroscopic disease seen at surgery and the resection length and found gastrointestinal involvement of >30 cm to be a statistically significant factor, while Fornaro [14] reported a length of intestinal disease of >100 cm to be a statistically significant factor. In our study, the length of intestinal disease was assessed both in radiological studies and surgical specimens. While the length of small-bowel pathological loops in radiological exams was not a statistically significant factor, we found both the sum of small-bowel pathological loops in radiological exams and the length of the resected intestine measured intraoperatively to be statistically significant. In particular, we found a correlation between the probability of recurrence and the grade of length increase. To the best of our knowledge, this correlation has not been verified in previous studies.

Finally, in our study, the presence of granulomas and hand-sewn anastomosis were statistically significant factors. In patients with granulomas, the median time from ileocolic resection to clinical postoperative recurrence was 13.4 months. However, the literature data regarding the impact on postoperative recurrence of the presence of granulomas in the resected specimen are contradictory. Some authors reported an association with a higher incidence of recurrence [18,19], while others reported a lower recurrence rate [20,21].

The result of an increased risk of recurrence in the case of hand-sewn anastomosis is a controversial fact that requires further investigation. In our opinion, although statistical significance was reached in our study, the result was influenced by the non-homogeneity of the numbers in the two groups (mechanical and hand-sewn anastomoses) and by the fact that, often, in our center, the decision to perform a mechanical anastomosis is influenced by the intraoperative characteristics, and, in the most difficult cases (fistulizing disease, abscesses, and extensive resections), a manual anastomosis is, generally, opted for.

The other surgical factors were not statistically significant factors.

### 4.5. Postsurgical Evaluation

The presence of complications was also not a statistically significant factor for recurrence.

In our study, we evaluated the risk of recurrence within two years of surgery and excluded patients who had already undergone surgery. On the contrary, Khoury et al. [5] evaluated the risk factors associated with early disease recurrence with the need for re-surgery and found that the risk factors for early disease recurrence were the presence of stenotic and penetrating disease (stricturing: odds ratio (OR)—12.1; penetrating: OR—9.9 (rather than non-stricturing and non-penetrating)) and the development of postoperative complications in a previous surgery.

### 4.6. Limitations

Our work presents several limitations that must be taken into consideration.

First and foremost, this was a retrospective study, and the validity of our conclusions is naturally influenced by this study’s design. Additionally, the postoperative recurrence of Crohn’s disease can be significantly affected by the postoperative pharmacological therapy and the timing of its initiation. Unfortunately, the data we had only provided partial information regarding the postoperative therapy taken by the patients in our study, which is why we chose not to include these data in our analysis. Certainly, this information could be incorporated into the design of a larger prospective study in the future.

## 5. Conclusions

Our study identified clinical, surgical, and radiological factors that could predict disease recurrence after intestinal resection for Crohn’s disease. Younger patients with disease localization in the distal ileum, extensive radiological involvement of small-bowel loops, and specific histopathological features, such as granulomas and longer resected segments, represent a high-risk group for recurrence. Identifying these factors preoperatively allows for the possibility of more aggressive therapeutic interventions post-surgery, including, for example, the early introduction of immunosuppressive or biological agents, which may mitigate the risk of recurrence and improve long-term outcomes. These results not only offer valuable insights into patient-specific recurrence risks but also set the stage for future prospective studies aimed at optimizing postoperative management and improving the quality of life of patients with Crohn’s disease. By refining our ability to predict recurrence, we can move closer to more personalized, precision-based treatment paradigms that address both the immediate postoperative period and long-term disease control.

## Figures and Tables

**Table 5:** Localizations of pathological intestinal loops in CT-E and MR-E.

	Number of Patients	Percentage (%)
Proximal jejunum	1	1.6
Distal jejunum	5	7.9
Proximal ileum	6	9.5
Distal ileum	41	65.1
Last ileal loop	62	98.4
Involvement of other intestinal loops:		
Appendix	16	25.4
Ascending colon	11	17.5
Transverse colon	3	4.8
Distal colon	2	3.2
Sigma	6	9.5
Rectum	3	4.8

**Table 6:** CT/MRI small-bowel pathological loops.

	Minimum	Maximum	Mean	Standard Deviation
Wall thickening (mm)	5	25	10.8	2.9
Lumen diameter (mm)	1	7	2.6	1.3
Pre-stenotic loop caliber (mm)	13	95	28.5	13.7

**Table 7:** MR-E, CT-E, and CT findings (35 MR-E, 15 CT, and 13 E-CT).

	Number of Patients	Percentage (%)
Mural thickening	63	100
Mucosal ulcers	27	42.9
Stratified CE	63	100
Halo fatty sign	3	4.8
Engorgement of vasa recta	59	93.7
Stenosis	60	95.2
Pre-stenotic dilatation	32	50.8
Fibrofatty proliferation	8	12.7
Sinus tracts	23	36.5
Enterocutaneous fistulas	5	7.9
Entero-enteric fistulas	25	39.7
Abscesses	15	23.8
LFN	54	85.7
Occlusion	8	12.7
MRI hyperintensity	35	97.2
MRI DWI restriction	30	85.7

**Table 8:** Extension of small-bowel disease in CT and MR exams.

	Minimum	Maximum	Mean	Standard Deviation
Extension of each pathological loop (mm)	20	600	186.1	120.4
Sum of the length of pathological loops (mm)	20	881	258.9	167.5

**Table 9:** Surgical potential risk factors for postoperative recurrence.

	Number of Patients	Percentage (%)
Type of surgical resection:		
Ileocecal resection	48	76.2
Ileocolic resection	14	22.2
Right hemicolectomy	1	1.6
Laparoscopic surgery	46	73
Emergency surgery	1	1.6
Histological involvement of resection margins	23	36.5
Type of anastomosis:		
Stapled anastomosis	11	17.5
Hand-sewn anastomosis	52	82.5
Presence of granulomas in the specimen	4	26.7
Transfusions	2	3.2
Ileostomy/colostomy	4	6.3

**Table 10:** Postsurgical complications and related Clavien–Dindo score.

	Number of Patients	Percentage (%)
Postoperative complications:	22	35
Anastomotic dehiscence	2	3
Sepsis	1	1
Fever (>37.5°)	9	9
Intra-abdominal fluid collection	3	5
Urinary tract infection	3	5
Anemization	5	8
Melena/rectorrhagia	9	14
Vomiting	1	1
High stomal output	1	1
Bladder globe	1	1
Hypoadrenal crisis	1	1
Resumption of parenteral nutrition	1	1
Delay of canalization	0	0
Clavien–Dindo grade:		
0	41	65
1	12	19
2	7	11
3a	1	2
3b	2	3

## Data Availability

The raw data supporting the conclusions of this article will be made available by the authors upon request.
